# Hydrophilization and Functionalization of Fullerene C_60_ with Maleic Acid Copolymers by Forming a Non-Covalent Complex

**DOI:** 10.3390/polym16121736

**Published:** 2024-06-19

**Authors:** Nadezhda A. Samoilova, Maria A. Krayukhina, Zinaida S. Klemenkova, Alexander V. Naumkin, Michail I. Buzin, Yaroslav O. Mezhuev, Evgeniy A. Turetsky, Sergey M. Andreev, Nelya M. Anuchina, Dmitry A. Popov

**Affiliations:** 1 A. N. Nesmeyanov Institute of Organoelement Compounds, Russian Academy of Sciences, 28 Vavilov St., 119334 Moscow, Russia; samoilova.nadezhda@gmail.com (N.A.S.); kmalex@yandex.ru (M.A.K.); zklem@ineos.ac.ru (Z.S.K.); naumkin@ineos.ac.ru (A.V.N.); buzin@ineos.ac.ru (M.I.B.); 2 Department of Biomaterials, Mendeleev University of Chemical Technology of Russia, 9 Miusskaya Square, 125047 Moscow, Russia; 3 NRC Institute of Immunology, FMBA, 24, Kashirskoye shosse, 115478 Moscow, Russia; e.turetskiy@gmail.com (E.A.T.); andsergej@yandex.ru (S.M.A.); 4 A. N. Bakulev National Medical Research Center for Cardiovascular Surgery of the Ministry of Health of the Russian Federation, 135 Rublevskoe Sh., 121552 Moscow, Russia; anuchinanelya@mail.ru (N.M.A.); da_popov@inbox.ru (D.A.P.)

**Keywords:** fullerene C_60_ aqueous dispersions, maleic acid copolymers, C_60_ nanocomposites

## Abstract

In this study, we report an easy approach for the production of aqueous dispersions of C_60_ fullerene with good stability. Maleic acid copolymers, poly(styrene-*alt*-maleic acid) (SM), poly(N-vinyl-2-pyrrolidone-*alt*-maleic acid) (VM) and poly(ethylene-*alt*-maleic acid) (EM) were used to stabilize C_60_ fullerene molecules in an aqueous environment by forming non-covalent complexes. Polymer conjugates were prepared by mixing a solution of fullerene in N-methylpyrrolidone (NMP) with an aqueous solution of the copolymer, followed by exhaustive dialysis against water. The molar ratios of maleic acid residues in the copolymer and C_60_ were 5/1 for SM and VM and 10/1 for EM. The volume ratio of NMP and water used was 1:1.2–1.6. Water-soluble complexes (composites) dried lyophilically retained solubility in NMP and water but were practically insoluble in non-polar solvents. The optical and physical properties of the preparations were characterized by UV-Vis spectroscopy, FTIR, DLS, TGA and XPS. The average diameter of the composites in water was 120–200 nm, and the ξ-potential ranged from −16 to −20 mV. The bactericidal properties of the obtained nanostructures were studied. Toxic reagents and time-consuming procedures were not used in the preparation of water-soluble C_60_ nanocomposites stabilized by the proposed copolymers.

## 1. Introduction

Fullerene C_60_, a spherical cage-shaped molecule, is one of the most studied in the family of carbon allotropes. Due to its exceptionally high symmetry, C_60_ is highly stable, and its structure is the most thermodynamically efficient. The molecule in the shape of a truncated icosahedron has a diameter of 0.7 nm and contains 12 pentagonal faces and 20 hexagonal ones. The electronic structure of fullerene determines its unique properties: electron acceptor activity, high polarizability, radical scavenging activity and, vice versa, a generation of free radicals under light irradiation. In addition, C_60_ has a large surface area with a large number of equivalent reaction sites [1]. Pure fullerene (pristine C_60_) and a number of its derivatives are commercially available products [2]. Fullerenes have been used as building blocks for the creation of covalent or non-covalent 2D/3D carbon materials (third-generation solar cells); such materials have also found application in catalysis, spintronic, water treatment, etc. [3,4,5,6]. The presence of C_60_ molecules leads to an increase in the photoconductivity of conducting polymers and organo-metallic compounds [7]. Due to their spherical form and unique electron properties, fullerenes attract researchers in the fields of medicine and biology [8,9,10,11]. Nanomaterials based on C_60_ have good prospects in cancer therapy [12,13,14]. Under the influence of UV radiation, fullerene derivatives (malonates) can cause HeLa cells’ death [15]. C_60_ fullerene compounds can provide radioprotection to radiosensitive mammalian cells [16]. A noticeable anti-inflammatory effect of an aqueous dispersion of C_60_ fullerene, which also acts as an antioxidant and an anti-aging antioxidant drug, has been shown [17,18,19,20]. The antibacterial properties and virucidal activity of fullerene and its derivatives have been described [21,22,23,24,25]. Fullerenes are able to reduce the apoptosis of neurons induced by reactive oxygen species (ROS). It is also assumed that by inhibiting the level of ROS, fullerenes can have an antiallergic effect [26]. However, none of the listed applications of aqueous and oil C_60_ dispersions are currently available to consumers, except skin creams [27]. A number of studies show that aqueous dispersions of pristine C_60_ do not possess acute or subacute toxicity [17,28,29,30].

For the use of C_60_ and its derivatives for biomedical purposes, it is preferable to disperse them in aqueous media due to the biocompatibility, non-toxicity and environmental friendliness of this solvent. The main approaches to the production of water-dispersible fullerene can be distinguished:

1. chemical modification of fullerene (derivatization),
2. direct dispersion in water and the solvent-exchange method,
3. non-covalent complexation of fullerene with a number of hydrophilic compounds,
4. encapsulation.

The most popular chemical modifications of fullerene are reactions of nucleophilic and radical additions, including cycloadditions, hydroxylation (fullerenol), carboxylation and amination (amino acid-containing adducts) [31,32,33]. The synthesis of covalent assemblies consisting of inherently chiral open inter–60 fullerenes has been demonstrated [34]. Fullerene molecules can also be incorporated into polymeric structures by covalent bonding. These include “pearl necklace” structures, “charm bracelet” structures, organometallic polymers, cross-linked polymers, end-capped polymers, star-shaped polymers and supramolecular polymers. Some of them have been shown to be soluble in water [35]. In cases of chemical modification, in order to give fullerene water-solubility, it is often necessary to introduce more than one functional group into its structure. Such fullerene derivatives are difficult to identify and separate because the system often contains a mixture of regioisomers. Such modification can change the chemical and physical properties of C_60_ molecules. In addition, methods of chemical C_60_ functionalization require harsh conditions, the use of catalysts or strong oxidizing agents, favoring cage opening.

The most striking individuality of fullerene molecules is manifested in an unmodified form. The dispersion of the fullerene in water was carried out in a series of experiments. At the same time, the prolonged mixing and sonication of fullerite and water were used [36,37,38]. Known methods for the formation of stable aqueous dispersions are based on the transfer of C_60_ from an organic solution (benzene, toluene, tetrahydrofuran) into an aqueous phase (“solvent exchange method”) using ultrasonic treatment. The organic solvent is gradually displaced by intense ultrasound, which ensures the heating of the system and the evaporation of the solvent [39,40]. Such dispersions contain hydrated clusters of C_60_ molecules; their size depends on the characteristics of the method. However, an aromatic solvent is not suited for obtaining solutions for medical purposes, and it is also difficult to standardize ultrasonic technology, which depends on the sonication time, volume and geometry of the vessel with the dispersion. It is also difficult to completely remove traces of toluene due to its specific π-stacking interaction with fullerene molecules [41]. The above approach is quite labor- and energy-intensive, and the final concentration of fullerene is limited (<10^−5^ M). It should be added that the dried C_60_ dispersions obtained by the abovementioned methods usually irreversibly lose their solubility in water.

An alternative approach for avoiding some of the above-stated problems is the conjugation of fullerene with a number of hydrophilic compounds without forming covalent bonds. The electron structure of fullerene is less affected during such bonding; hence, its physical properties are retained to a significant extent. Some variants are the supramolecular encapsulation of fullerene with γ- or β-cyclodextrin and dendritic derivatives of cyclotriveratrilene and calixarenes [42,43,44,45]. However, the strength of such complexes does not always correspond to the required one. The solubilization of fullerene with proteins is another option [46,47]. The encapsulation of fullerene C_60_ in lipid micelles has been described by many researchers [48,49,50,51]. Although nonionic surfactants such as Triton and Twin provide the good solubilization of C_60_, this method also uses toluene to initially dissolve the fullerene, which then must be removed [49].

The approach of including fullerene in polymers seems promising. Supramolecular chemistry approaches for the preparation of host–guest inclusion complexes with fullerenes have made significant progress over the past decade [3]. Polymer-fullerene nanocomposites are new materials with many applications in the biomedical field [8]. Fullerenes can be retained in the cavity of host polymer molecules due to van der Waals and hydrophobic interactions and π-π stacking, as well as electrostatic interactions [52]. It is known that polymers involving oxygen or (and) nitrogen (electron-donor elements) may form charge-transfer complexes with fullerene [53,54]. The inclusion of fullerene in the compositions of hydrophilic polymers can provide the ability for them to be dispersed in water; in addition, the manufacturability of such complexes increases, which facilitates their use in a variety of fields. The dispersibility of C_60_ in polar solvents may increase using solubilization with amphiphilic polymers, such as polyethylene glycol [55], poly(styrene-b-dimethylacrylamide) block copolymer [56] or poloxamer [10]. Water-soluble polymers of acrylamide and acrylic acid that contain fullerene have been prepared by the low-temperature radiation-induced living polymerization in organic solvents [57]. The most well-known are water-soluble fullerene complexes with polyvinylpyrrolidone (PVP) [58,59,60,61,62]. During the complexation of fullerene with PVP, the fullerene content in relation to the polymer usually does not exceed 1–1.5% [60].

Maleic acid copolymers have not yet been used for the hydrophilization of fullerene. Meanwhile, such copolymers have a number of positive properties: they are water-soluble and amphiphilic, and many of them are commercially available and nontoxic. The copolymers have a regular structure—polymer units clearly alternate. Previously, such copolymers were used to stabilize silver nanoparticles [63], colloidal nanohybrid structures of silver (gold) and zinc oxide [64,65]

In this work, we propose to use maleic acid copolymers for the hydrophilization of fullerene and the introduction of easily modified functional groups into the composition. The complexation of fullerene C_60_ to copolymers was carried out using the so-called “dialysis principle” [66].

## 2. Materials and Methods

### 2.1. Materials

Poly(N-vinyl-pyrrolidone-*alt*-maleic anhydride) M = 40,000 was prepared according to [67]. Poly(styrene-*alt*-maleic anhydride) M = 50,000 and poly(ethylene-*alt*-maleic anhydride) with an average molecular weight of M = 25,000 were purchased from Sterlitamak chemical plant (Sterlitamak, Russia) and Monsanto (Saint Louis, MO, USA), respectively. The copolymers of maleic acid (VM, EM and SM) were obtained by the hydrolysis of the corresponding copolymers of maleic anhydride by dissolution in deionized water followed by lyophilization fullerene C_60_ (the van der Waals diameter is about 1.1 nm, 99.9%, SES Research, catalog 600–9969, Houston, TX, USA), N-methylpyrrolidone (NMP) (99%, Panreac AppliChem GmbH, Ottoweg 4, D-64291 Darmstadt, Germany) and NaOH (analytical grade, Reakhim, Moscow, Russia), which were used without purification. The 1 kDa dialysis tubes were from Spectra/Por (Spectrum Labs, Pittsburgh, PA, USA).

The following tested microorganisms were used (“BD MicrotrolTM”, Becton Dickinson, Franklin Lakes, NJ, USA): *Candida albicans* (*C. albicans*) NCPF3255/ATCC 10231, *Escherichia coli* (*E. coli*) NCTC 11954/ATCC 35218, *Pseudomonas aeruginosa* (*P. aeruginosa*) NCTC 12903/ATCC 27853, *Staphylococcus aureus* MRSA (*S. aureus MRSA*) NCTC 12973/ATCC 43300 and *Enterococcus faecalis* (*E. faecalis*) NCTC 12697/ATCC 29212.

### 2.2. Instrumentation

The pH values were determined using a Fisher Scientific 300 403.1 pH-meter (Waltham, MA, USA). FTIR spectra (KBr) were recorded using a Fourier-spectrometer Magna IR-720 (Nicolet, Parsons, WV, USA). The UV-visible absorption spectra were measured with a UVIKON-922 spectrophotometer (Zeiss Group, Baden-Württemberg, Germany). Registration was carried out without diluting the reaction solution, and a cuvette with a diameter of 0.2 cm was used for spectrophotometric measurements.

X-ray photoelectron spectroscopy studies were carried out on an Axis Ultra DLD spectrometer (Kratos Analytical, Manchester, UK) using monochromatic Al Kα (hν = 1486.6 eV) radiation. Survey spectra and high-resolution spectra were recorded at pass energies of 160 and 40 eV with steps of 1 eV and 0.1 eV, respectively. Survey and high-resolution spectra were recorded with steps of 1 eV and 0.1 eV, respectively. The sampling area was 300 × 700 μm^2^. Samples were mounted on a titanium holder using a double-sided adhesive tape and studied at room temperature under a vacuum of <10^−8^ Torr. The energy scale of the spectrometer was calibrated according to the standard procedure based on the following binding energies: 932.62, 368.21 and 83.96 eV for Cu 2p_3/2_, Ag 3d_5/2_ and Au 4f_7/2_, respectively. To eliminate the effect of sample charging, the spectra were recorded using a neutralizer. Surface charging was corrected by referencing to the C-C/C-H peak identified in the C 1sspectra (285.0 eV). The background due to electron inelastic energy losses was subtracted by the Shirley method. Quantification was performed using atomic sensitivity factors included in the software of the spectrometer.

The elemental analysis was performed using an Elementar Vario MICRO cube C, H, N-analyzer (DKSH Group, Tokyo, Japan) equipped with a thermal desorption column and a UV-Vis spectrophotometer (Agilent Technologies, Santa Clara, CA, USA).

Measurements of ξ-potential and nanoparticle sizes were performed using dynamic light scattering (DLS) on a Zeta Sizer Nano ZS instrument (Malvern Instruments, Worcestershire, UK). The thermal stability of the initial copolymers, the copolymer and C_60_ mixture and the composites was studied by thermogravimetric analysis (TGA). TGA measurements were performed by a Derivatograth-C (MOM Szerviz, Budapest, Hungary) on samples of about 15 mg at a heating rate of 10 °C/min in argon.

### 2.3. Methods

#### 2.3.1. Synthesis C_60_/Copolymer Composites

Synthesis of C_60_/VM: Initially, 6.25 mL of solution crystalline C_60_ in NMP (0.8 mg/mL) was mixed with 4 mL of an aqueous solution of VM (2 mg/mL, pH 8) using a magnetic stirrer. The molar ratio of the maleic acid residues of copolymer to the mole C_60_ was 5/1. After 0.5 h, the resulting solution was subjected to exhaustive dialysis (cutoff 1 kDa) against deionized water (1.5 L, four changes). The dried sample was obtained with subsequent lyophilic drying (−55 °C, 0.05 mbar). The samples C_60_/SM and C_60_/EM were prepared under similar reaction conditions and reagent concentrations; the molar ratios of monomeric units of maleic acid residues of the copolymer to C_60_ were ~5/1 and 10/1, respectively.

#### 2.3.2. Antimicrobial Activity Tests

The method of serial microdilution was used for the determination of the minimum inhibitory concentrations (MIC) of the preparations in relation to the strains of microorganisms in accordance with the standard procedure [68]. The initial concentrations of C_60_/VM and C_60_/SM were 0.46 and 0.37 mg/mL, respectively (C_60_/EM—0.50 mg/mL). A detailed description of the experiment was given elsewhere [69].

## 3. Results

To obtain a stable colloidal solution of a fullerene–polymer complex, the choice of a polymer component (matrix) as a stabilizer is very important. To prevent the aggregation of fullerene nanoparticles by reducing their surface energy, the choice of a suitable polymer coating of nanoparticles is very important. The stabilization of fullerene nanoparticles can occur not only due to hydrophobic and/or π-π interactions; spatial and/or Coulomb stabilization are also significant. The selected amphiphilic maleic acid copolymers are suitable according to the above criteria. NMP was selected as an aprotic solvent in which fullerene C_60_ (fullerite) and polymers are sufficiently soluble due to its low toxicity (taking into account the medical aspect of this work) [70]. In a further description of the processes for obtaining stabilized aqueous dispersions of C_60_ and an analysis of their properties, for the purposes of the present article, the term “dissolution” will be used, with the resulting system being a colloidal solution (nanodispersion).

**Figure 1:**
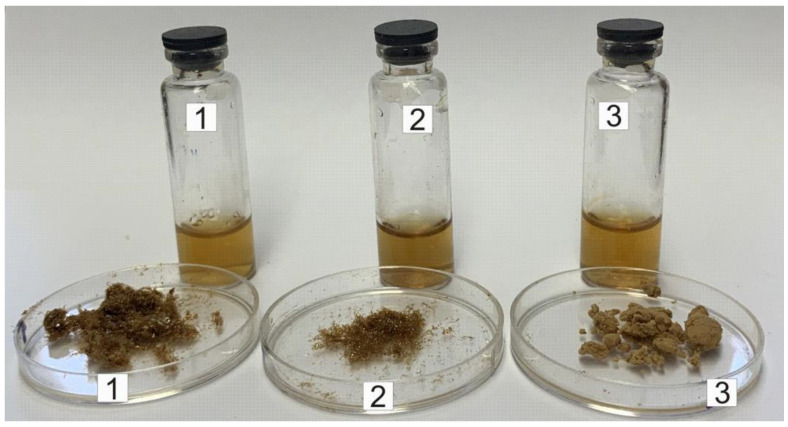
Aqueous solutions and dry forms of polymer–fullerene samples: 1—C_60_/SM, 2—C_60_/EM, 3—C_60_/VM.

Polymer-stabilized fullerene complexes can be obtained in two main ways: the dissolution (co-dissolution) of the fullerene and copolymer in NMP or the addition of an aqueous polymer solution to a fullerene solution in NMP. The second variant is preferable because it takes less time and the final solution contains less NMP. Meanwhile, the co-dissolution of the fullerene and polymer in water at pH 6–8 did not result in a homogeneous solution. We used an approximately fivefold molar excess of the VM and SM copolymers relative to fullerene and a tenfold excess for the EM copolymer (calculated per dimer unit), which corresponded to a 1.6–2.0-fold excess by weight. The calculated content of fullerene in the samples was 33–38%. The values found according to the elemental analysis, gravimetric data and spectrophotometry of the corresponding solutions (λ = 340 nm, ε = 68,000 dm^3^ moL^−1^ cm^−1^) [71] were 30–33%. The increase in the fullerene content relative to the copolymer resulted in a stronger processes of aggregation in a solution and was accompanied by the appearance of large particles separated into the solid phase. The volume ratio of the NMP solution to water was equal to 1:1.2–1.6. The resulting polymer-fullerene solutions were subjected to exhaustive dialysis (cutoff 1 kDa) against deionized water to remove the organic solvent. Drying the yellow–brown dialysate yielded brown powders (Figure 1) that were soluble in water. According to XPS and elemental analysis, the product contained approximately 1–2% nitrogen, even in the case of composites with nitrogen-free copolymers. This indicated that the products contained solvated NMP, and this was not due to insufficient dialysis, since fullerene forms charge transfer complexes with NMP [38,72,73]. According to the elemental analysis data, the conjugates contained approximately 5–10% water. It is known that fullerene is also capable of complexing water [74].

**Figure 2:**
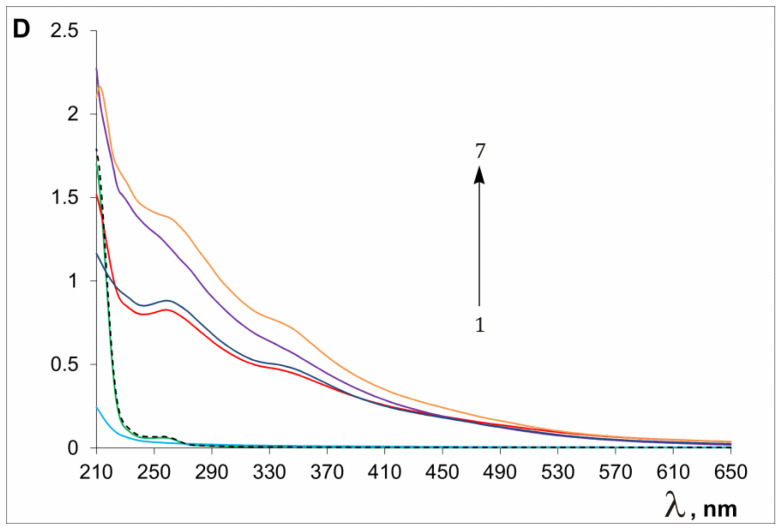
Absorption spectra of water solutions of copolymers and C_60_ conjugates: 1—EM, 2—SM, 3—VM; 4—C_60_/SM, 5—C_60_/VM, 6—C_60_/EM; (C = 80–90 μg/mL, l = 0.2 cm); 7—C_60_/VM after drying and dissolving (C = 100 μg/mL).

After dialysis, the concentration of the complexes in an aqueous solution was 300–400 μg/mL (fullerene content—50–100 μg/mL). The stability of the aqueous solutions of the composites, according to our estimates, is at least 12 months when stored at 4–25 °C. The fullerene composite containing the most hydrophilic VM copolymer (C_60_/VM) had the best solubility—approximately 500 μg/mL at pH 7.0. The solubilities of C_60_/SM and C_60_/EM were noticeably worse—about 100 and 50 μg/mL, respectively. The solubility of the complexes in NMP was approximately 300–400 μg/mL for C_60_/SM and C_60_/VM and 500 μg/mL for C_60_/EM. Fullerene C_60_ is practically impossible to extract from dried composites with chloroform, benzene and toluene, and no extraction of C_60_ occurred from water solutions of composites with the use of such solvents. So, these complexes practically do not break down in these solvents. Figure 2 shows the UV-Vis spectra of the initial copolymers and obtained conjugates. Solutions of the copolymers VM, EM and SM are colorless and almost do not absorb at wavelengths greater than 300 nm (Figure 2(1–3)).

The absorption spectrum of fullerene C_60_ in nonpolar hexane (where its aggregation is minimal) is characterized by four maxima at 213, 257, 328 and 406 nm [75]. In our case, the spectra of the composites do not have such distinct maxima (Figure 2(4–7)); there are small hills in the region of 260 and 340 nm. This is probably due to the self-association of fullerene molecules, aggregation with copolymers, binding to NMP and water molecules and also some loss of the icosahedral symmetry of C_60_ molecules [76]. After several months of storage, the spectra of the obtained solutions did not change.

The hydrodynamic particle size of the diluted C_60_ samples measured by DLS ranged from 116 to 200 nm, with a polydispersity index (PDI) of 0.20–0.40 (Table 1). The distributions of scattered-light intensity over the particle size in the solutions of the hybrid macromolecular structures C_60_/SM and C_60_/VM were monomodal.

**Figure 3:**
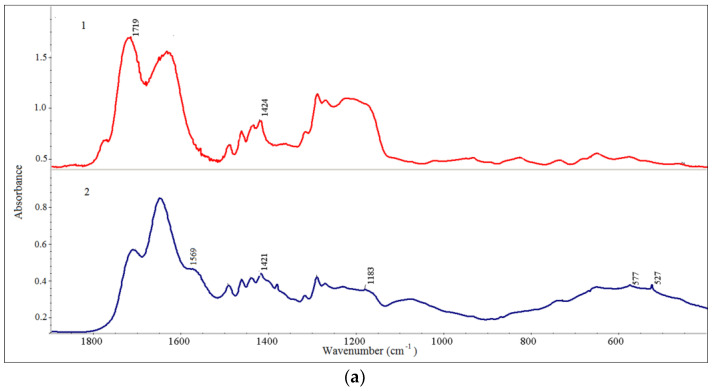

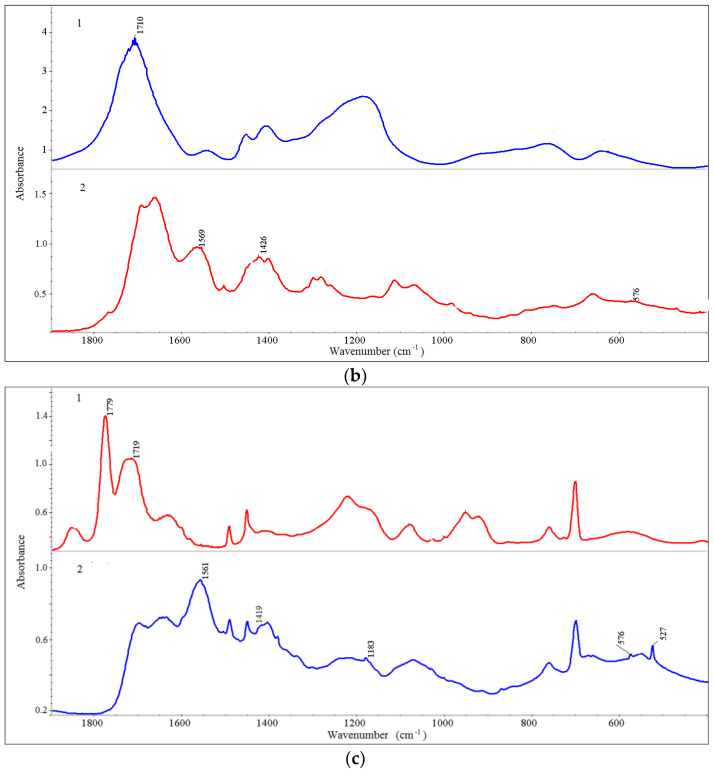
FTIR spectra of samples containing copolymers: (**a**) VM, (**b**) EM and (**c**) SM; initial copolymers (**a1**–**c1**) and fullerene conjugates (**a2**–**c2**).

The size distributions of composite particles are different and are related to the structure of polymer chains. When using a copolymer EM as a stabilizer C_60_, the distribution was bimodal, and the colloidal solution contained micron-sized particles. The presence of fullerene in composites apparently provides the greatest contribution to the aggregation of the resulting hybrid structures. The composite particle size may be related to the homo aggregation of fullerene nC_60_ ↔ (C_60_)n [77,78,79]. At the same time, it was previously shown that maleic acid copolymers can aggregate in acidic and alkaline media, and the particle size distributions were bimodal, with the fast mode correlated with single chains (2–4 nm) and the slow mode correlated with polymer aggregates (60–120 nm). Meanwhile, EM copolymers aggregate to a noticeably greater extent [80,81]. Apparently, the polymer acts as a hydrophilic shell on the surface of fullerene aggregates. The used copolymers involve electron-donor elements, oxygen and nitrogen (in the case of VM), so they may form charge-transfer complexes with fullerene [53]. It can be assumed that the SM copolymer is able to bind to fullerene through π-π interactions. Since C_60_, toluene and xylene contain sp^2^-states, C_60_ acts as a π-electron acceptor when interacting with them [82]. Besides donor–acceptor interactions, Coulomb stabilization plays a role in the stabilization of the colloid solution of complexes. The copolymers of maleic acid contain two carboxyl groups with different pK values in the monomeric unit. We have shown previously [83] that in the pH range of 4–6, one carboxyl group of the maleic acid residue in copolymers was ionized by 60–70%, while the other was practically not ionized; however, at a neutral pH, the ionization of both carboxylic groups will be significant. The ξ-potential measured for all samples was from −16 to −20 mV. The obtained values of the zeta potentials indicate the good stability of the colloidal solution of the composites [84]. A negative zeta potential means that the composite surface has a negative charge, which may contribute to the high dispersion stability in the solution due to the inter-particle electrostatic repulsive forces. The FTIR spectra of the fullerene composites and initial copolymers are presented in Figure 3.

The characteristic absorption bands of fullerene C_60_ at 1429, 1181, 576 and 527 cm^−1^ are attributed to the C-C vibrational modes of the C_60_ molecules [85]. Against the background of the absorption bands of the copolymers in the IR spectra of hybrid structures, characteristic absorption bands of fullerene are observed at ν = 527, 576 (577) and 1419 (1421, 1426) cm^−1^ (Figure 3(a2–c2)). Due to the overlap of the polymer and fullerene bands, Figure 3 highlights the most intense bands of fullerene. This indicates the presence of native fullerene in the C_60_–copolymer conjugates. The noticeable change in the FTIR spectra between the composites and copolymers is due to the position of the C=O bond of the carboxyl group. For example, in the VM spectrum (Figure 3(a1)), C=O stretching vibration at 1719 cm^−1^ corresponds to non-ionized carboxyl groups of maleic acid residues. In the FTIR spectrum of C_60_/VM (Figure 3(a2)), the value C=O stretching vibration is shifted to the region at 1575–1576 cm^−1^, which corresponds to the ionized form of the carboxyl group of the maleic acid residues of the copolymers [65]. For the composites C_60_/EM and C_60_/SM, this band is in the range of 1561–1569 cm^−1^. The sample SM (Figure 3(c1)) also contained partially non-hydrolyzed anhydride groups (bonds 1779, 1860 cm^−1^), which, in the composite, were also converted into ionized carboxyl groups.

**Figure 4:**
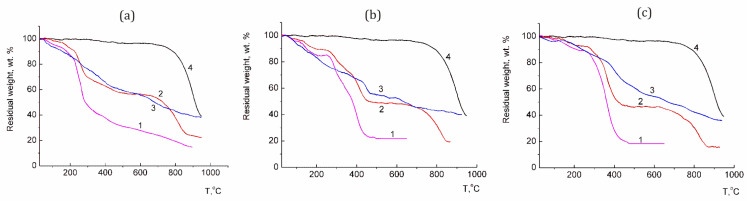
TGA curves of samples containing VM (**a**), EM (**b**) and SM (**c**) copolymers: 1—copolymers, 2—mechanical mixtures of copolymer with C_60_, 3—hybrid structures and 4—C_60_. Heating rate of 10 °C/min (argon atmosphere).

A comparative analysis of the TGA curves of hybrid structures C_60_/copolymer, _C60_, copolymers and a mechanical mixture of copolymers with fullerene can serve as proof of the inclusion of fullerene in the structure of the polymer composite (Figure 4).

The native C_60_ was stable up to 800 °C, with almost no loss of mass (Figure 4a–c, curves 4). The initial copolymers decompose mainly at about 400 °C (Figure 4a–c, curves 1). The TGA curves of mechanical mixtures (~30 wt.% fullerene) practically repeat the curves of the individual components contained in the mixture. The decomposition degree of the fullerene-containing samples occurred at about 100 °C lower than that of individual C_60_. The TGA curves of C_60_–copolymer hybrid structures (Figure 4a–c, curves 3) differ from those of mixtures of system components in a smoother course. At low temperatures, the samples lost mass, probably as a result of the cleavage of solvent molecules. Table 2 shows comparative data on the decomposition of the studied samples.

**Figure 5:**
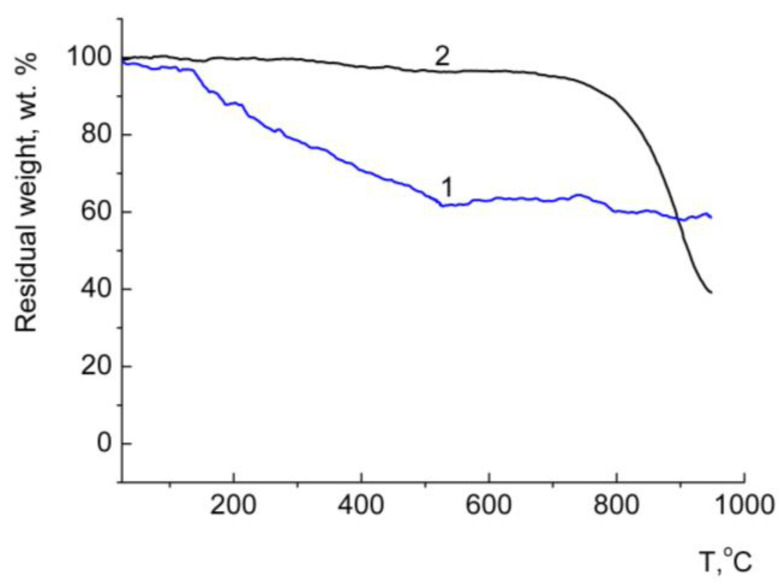
TGA curves: 1—C_60_ after dialysis and lyophilic drying, 2—pristine C_60_.

We noted that, for composites, the last stage of destruction lasts longer and above 700 °C, in contrast to the mechanical mixtures and initial copolymers (Figure 4, Table 2). Previously, a similar effect was observed for C_60_/epoxy polymer nanocomposites [86]. For composite samples, mainly, the found values of the residual weight at 600 °C exceeded those for the calculated values (Table 2). We conducted a control experiment in which fullerene was subjected to the same procedure that was used in the preparation of conjugates: the C_60_ solution in NMP was subjected to dialysis against water, followed by lyophilic drying. The resulting water-insoluble red–brown powder was also studied by the TGA method (Figure 5, curve 1).

The TGA curve for this compound differs from that of the original C_60_, and its tendency toward decomposition is similar to that of C_60_ complexes with copolymers. In this case, the last stage of destruction of this sample also continues at temperatures above 700 °C; the decomposition stage of fullerene at this temperature is practically degenerate. In contrast to the C_60_ fullerene, at temperatures above 100 °C, a loss of mass is observed, probably due to the removal of bound water and NMP. Judging by the TGA curves, the composites do not include individual fullerene formations, and obviously, the fullerene component is associated with NMP molecules, which was also confirmed by DLS, elemental analysis and XPS data.

**Figure 6:**
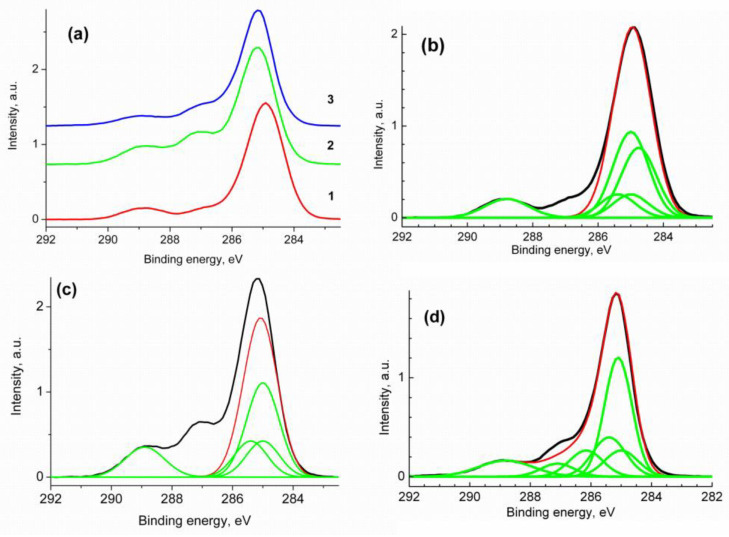
The high-resolution C 1s spectra normalized by the intensity of the main peak ((**a**) 1—C_60_/SM, 2—C_60_/EM, 3—C_60_/VM), sample C_60_/SM (**b**), sample C_60_/EM (**c**) and sample C_60_/VM (**d**). Black—experimental line, green—Gaussian profiles used for fitting, red—result of fitting.

Figure 6 shows the high-resolution C 1s photoelectron spectra of the prepared composites. The spectra in Figure 6a are normalized by the intensity of the main peak. Figure 6b–d show the spectra of the samples C_60_/SM, C_60_/EM and C_60_/VM fitted with several Gaussian profiles in accordance with their chemical formulas and reliable chemical shifts, respectively [87].

The characteristics of the peaks are presented in Table 3. Based on the quantitative analysis data, the binding energies and Figure 6, it can be concluded that NMP impurity is present in all samples, mainly in the C_60_/EM sample. The surface elemental compositions of the samples C_60_/SM, C_60_/EM and C_60_/VM calculated from high-resolution XPS spectra are C_80.4_O_18.2_Na_0.5_N_0.9_, C_76.6_O_21.4_Na_0.7_N_1.3_ and C_82.4_O_15.2_Na_0.3_N_2.1_, respectively.

The relative concentrations of C_60_ decrease in the sequence C_60_/EM, C_60_/VM and C_60_/SM (Table 3). The -C=C- groups correspond to the benzene ring in the C_60_/SM sample. CH_2_ groups are present in all carbochain copolymers, as well as carboxyl groups of maleic acid residues -C(O)O (Table 3). Thus, an analysis of XPS spectra confirms the presence of fullerene in the resulting conjugates, as well as the composition of the samples.

The resulting composites were tested for antibacterial activity against conditionally pathogenic microorganisms by the serial microdilution method. We use *E. coli*—Gram-negative, facultative anaerobic bacteria; *S. aureus MRSA*—Gram-positive bacteria, resistant to oxacillin; *C. albicans*—yeast-like fungi; *P. aeruginosa*—aerobic non-fermenting Gram-negative bacteria and *E. faecalis*—Gram-positive, commensal bacterium as test microorganisms. At a sample concentration of 300–400 μg/mL, the bactericidal activity of the studied conjugates was not detected against all the strains of the microorganisms used. When using an increased concentration of C_60_/VM up to 400 μg/mL, no activity against *C. albicans* was also observed. It may be noted that Japanese researchers showed carboxyfullerene (six carboxyl groups attached to fullerene C_60_) to be effective in the treatment of both Gram-positive and Gram-negative infections in in vitro experiments in doses of 5–50 μg/mL [21]. At the same time, C_60_ fullerene in the form of a complex with polyvinylpyrrolidone does not affect the microflora as well as any of the tested microorganisms, including Gram-positive cocci, streptococci, enterococci and Gram-negative bacteria [22]. Although the resulting stabilized aqueous C_60_ dispersions did not exhibit antibacterial activity, their subsequent modification makes it possible to obtain a sample with a pronounced antimicrobial effect. When silver nanoparticles in complex with EM (EM/Ag^0^) were introduced into the composite with fullerene, the MIC value in relation to *S. aureus* MSSA was 19.5 μg/mL or 6.7 μg/mL in terms of the silver content in the sample. For EM/Ag^0^, the MIC value is 54.5 μg/mL or 23.4 μg/mL in terms of the silver content in this sample. Thus, the resulting aqueous C_60_ dispersions, on the one hand, are promising as potential non-toxic carriers of pharmacologically active substances, and on the other hand, they can be modified to impart antibacterial activity.

## 4. Conclusions

We have demonstrated the production of stable water-soluble composites of C_60_ containing significant amounts of fullerene. Available ionogenic amphiphilic carbon chain copolymers of maleic acid of a regular structure were used for stabilization and hydrophilization agents in such systems. The protocol for the preparation of C_60_ conjugates eliminates the use of toxic organic solvents and time-consuming methods for their preparation, such as heating and prolonged stirring, ultrasonic treatment, etc. Such transformation is achieved under mild conditions when using the so-called “dialysis method” that is easily implemented. In a scalable version, a similar process, tangential ultrafiltration, can also be used. In the resulting nanostructures, guest molecules of C_60_ are confined by a polymer shell. The polymer shell provides the stabilization and solubility of encapsulated C_60_ in water through non-valent interactions—hydrophobic, steric and electrostatic. The resulting composites contained about 30% fullerene. The composite containing a more hydrophilic copolymer—poly(*N*-vinyl-pyrrolidone-*alt*-maleic acid) had better solubility in water. Although the composites had no activity as antibacterial agents, their fortification by introducing active bactericides, such as silver, can impart antimicrobial properties. The proposed approach may be used to obtain composites containing modified fullerene—for example, its amino acid adducts and other derivatives, which exhibit biological activity but poor solubility. There is the potential for using the obtained fullerene conjugates in other fields. The antioxidant properties of fullerene as part of a complex with non-toxic copolymers can be manifested in the creation of external products and can be used in cosmetology and dermatology [88].

## Figures and Tables

**Table 1:** The size and ζ–potential of hybrid macromolecular structures measured by the DLS method.

Sample ^a^	Average Size (nm) ^b^	PDI	ζ (mV)
C_60_/SM	202.3	0.21	−19.5
C_60_/VM	155.0	0.40	−17.6
C_60_/EM	116.1; 1387	0.20; 0.40	−15.7

^a^ The sample concentration was 80–90 μg/mL in deionized H_2_O, T = 25 °C; ^b^ large particles were previously removed by filtration through a glass filter S2.

**Table 2:** Characteristics of the samples and TGA analysis data.

Sample ^a^	C_60_ (wt.%)		Residual Weight ^b^ (%)	
	Calculated	Found ^a^	Found	Calculated
VM	-	-	27	-
C_60_/VM complex	38	32	55	54
C_60_/VM mixture	38	-	55	55
EM	-	-	22	-
C_60_/EM complex	33	33	53	48
C_60_/EM mixture	32	-	49	47
SM	-	-	14	-
C_60_/SM complex	38	31	60	47
C_60_/SM mixture	37	-	45	46

^a^ The average values according to the methods of gravimetric and elemental analysis and spectrophotometry of the corresponding solutions (λ = 340 nm, ε = 68,000 dm^3^·mol^−1^·cm^−1^) [71]; ^b^ at 600 °C.

**Table 3:** Parameters of components in the C 1s photoelectron spectra of the studied samples: E_b_—binding energy, W—Gaussian peak width and I_rel_—relative intensity.

Sample	Group	-C=C-	CH_2_	C_60_	C*-C(O)	C-N	C(O)N	C(O)O
C_60_/SM	E_b_	284.76	285.0	285.0	285.4			288.0
	W	1.08	1.08	1.08	1.08			1.34
	I_rel_	0.31	0.10	0.38	0.10			0.10
C_60_/EM	E_b_		285.0	285.0	285.4			288.9
	W		1.08	1.08	1.08			1.3
	I_rel_		0.18	0.47	0.18			0.18
C_60_/VM	E_b_		285.0	285.0	285.4	288.16	287.1	288.8
	W		1.1	0.9	1.1	1.1	1.1	1.79
	I_rel_		0.11	0.43	0.17	0.11	0.06	0.11

* Carbon atoms bonded to C(O) group.

## Data Availability

The data presented in this study are publicly available.
